# Interaction network of African swine fever virus structural protein p30 with host proteins

**DOI:** 10.3389/fmicb.2022.971888

**Published:** 2022-08-15

**Authors:** Xiongnan Chen, Xiaojun Chen, Yifan Liang, Sijia Xu, Zhijun Weng, Qi Gao, Zhao Huang, Guihong Zhang, Lang Gong

**Affiliations:** ^1^Guangdong Provincial Key Laboratory of Zoonosis Prevention and Control, College of Veterinary Medicine, South China Agricultural University, Guangzhou, China; ^2^African Swine Fever Regional Laboratory of China, Guangzhou, China; ^3^Research Center for African Swine Fever Prevention and Control, South China Agricultural University, Guangzhou, China; ^4^National Engineering Research Center for Breeding Swine Industry, South China Agricultural University, Guangzhou, China; ^5^Maoming Branch, Guangdong Laboratory for Lingnan Modern Agriculture, Guangzhou, Guangdong, China; ^6^Key Laboratory of Animal Vaccine Development, Ministry of Agriculture and Rural Affairs, Guangzhou, Guangdong, China

**Keywords:** African swine fever virus, p30, membrane protein interaction, endocytosis, innate immunity

## Abstract

African swine fever virus (ASFV) is a complex nucleocytoplasmic large DNA virus (NCLDV) that causes a lethal hemorrhagic disease that is currently threatening the global pig industry. ASFV structural protein p30 is a membrane phosphoprotein that suggests it may play a regulatory role, possibly in signal transduction. Despite its significance in internalization into host cells, the interaction between p30 and host proteins is relatively unknown. In this study, we describe the application of a DUALmembrane yeast two-hybrid assay to screen a primary porcine alveolar macrophages cDNA library and analyze the interactome of p30 protein. Our data identify seven host cellular proteins (DAB2, RPSA, OAS1, PARP9, CAPG, ARPC5, and VBP1) that putatively interact with the p30. We further verified the interaction between p30 and host proteins by laser confocal microscopy, co-immunoprecipitation, and GST-pulldown assay. To further understand the relationship between host proteins and p30, we drew the interaction network diagram and analyzed the functional enrichment of each host protein. Enrichment analysis of Gene Ontology and Kyoto Encyclopedia of Genes and Genomes indicated that host proteins were mainly related to endocytosis, actin cytoskeleton regulation, and innate immunity. Collectively, we identified the interaction between p30 and host cell protein using a membrane protein yeast two-hybrid system, which increases our knowledge of the interaction between ASFV and the host and informs future research on antiviral strategies.

## Introduction

African swine fever (ASF) is a highly pathogenic infectious disease caused by the African swine fever virus (ASFV), which mainly infects wild boars and domestic pigs. It is the only member of the Asfarviridae family, with a mortality rate of 100% (Penrith et al., 2019; Blome et al., 2020). ASF was first identified in Kenya and eastern Africa in the early twentieth century, and the first outbreak of ASF was reported in China in 2018 (Zhou et al., 2018; Mushagalusa et al., 2021). Presently, ASFV has no effective commercial vaccine or antiviral drug and can only be strictly prevented and controlled. Once this disease occurs, only isolation and culling can prevent an epidemic.

ASFV is a large enveloped virus with icosahedral morphology, which has caused great economic loss to the pig industry in affected countries. The ASFV particle structure, from inside to the outside, comprises a nucleoid, inner core-shell, inner envelope, outer capsid, and outer envelope. The diameter of the complete virus particles is ~200–300 nm (Wang et al., 2019; Andres et al., 2020), and it relies on its huge genome structure to encode many proteins that participate in virus entry, replication, transcription, assembly, and immune escape (Alejo et al., 2018; Wang et al., 2021). Although the functions of some proteins have been widely studied, there are still many proteins whose functions are unknown.

ASFV p30 protein is encoded by the virus gene CP204L, and its relative molecular weight is about 30 kDa (Sanchez et al., 2013). It is expressed in the early stage of ASFV infection and is often used to detect antibodies in the early stage of ASF infection (Petrovan et al., 2019). It is also a membrane phosphorylated protein, which appears on the infected cell membrane in the early stage after infection, and experiments have confirmed that the protein can be secreted outside the cell during the infection process (Afonso et al., 1992). ASFV and poxvirus belong to nucleocytoplasmic large DNA viruses (NCLDV), and their encoded proteins are similar in function (Iyer et al., 2001). Previous studies have confirmed that the secreted protein of poxvirus is often related to the virulence of the virus (Kotwal and Moss, 1988; Kotwal et al., 1990). In addition, ASFV p30 can also be used as an essential protein involved in virus internalization into host cells. Previous studies have shown that pretreatment with anti-p30 antibodies can inhibit more than 95% virus internalization in porcine macrophages and Vero cells. However, neutralizing antibodies induced by p30 are not sufficient to provide effective immune protection (Gomez-Puertas et al., 1996). Although we understand part of the function of p30, the lack of information on the interaction between p30 and host cells may be limiting our understanding of this phenomenon.

A yeast two-hybrid system is an effective way to analyze protein interactions *in vitro*. It includes nuclear and membrane yeast two-hybrid systems (Chen and Wei, 2022). Early studies based on the nuclear yeast two-hybrid system method screened the interaction of heterogeneous nuclear ribonucleoprotein K (hnRNP-K) with ASFV p30, which may be involved in the downregulation of host cell mRNA translation after ASFV infection (Hernaez et al., 2008). However, ASFV p30 protein, as a membrane phosphorylated protein, cannot sufficiently explain the p30 membrane protein interaction by using the nuclear yeast two-hybrid system. Therefore, to further understand the membrane-related interacting proteins of ASFV p30 protein, we established a cDNA library using primary porcine alveolar macrophages (PAM) and screened PAM cell proteins interacting with ASFV p30 protein through the DUALmembrane yeast two-hybrid system. The screening results were verified by laser confocal microscopy, Co-IP, and glutathione S-transferase (GST) pulldown experiments. Subsequently, the protein interaction network (PPI), Gene Ontology (GO) enrichment, and Kyoto Encyclopedia of Genes and Genomes (KEGG) pathway analyses were mapped for these interacting proteins. These results provide a theoretical basis for further studies on the involvement of the mechanism of ASFV p30 in ASFV infection.

## Materials and methods

### Cells, virus, antibodies, and reagents

PK-15 and HEK-293 T cells were cultured in a DMEM containing 10% fetal bovine serum (Gibco, Grand Island, NY, United States) and incubated at 37°C in a 5% CO2 atmosphere. The high virulence, hemadsorbing ASFV isolate GZ201801 (GenBank: MT496893.1) was isolated in Guangzhou, China, is p72 genotype II, and is preserved in the Infectious Diseases Laboratory of South China Agricultural University. Mouse-derived p30 mAbs were prepared in our laboratory. Mouse anti-FLAG monoclonal antibody was purchased from Sigma (F1804, St. Louis, MO, United States). The mouse anti-HA monoclonal (M20003) and rabbit anti-HA polyclonal (TT0050) antibodies were purchased from Abmart (Shanghai, China). Rabbit anti-OAS1 polyclonal antibody (14955-1-AP) were purchased from ProteinTech Group (ProteinTech Group, Chicago, IL, United States). The mouse anti-GADPH monoclonal antibody (HC301-01) was purchased from TransGen (Beijing, China). Goat anti-mouse IgG (H + L) Alexa Fluor Plus 488 secondary antibody was purchased from Thermo Fisher Scientific (A-11001, Waltham, MA, United States). IRDye^®^ 800CW goat anti-mouse IgG (926–32210) and IRDye^®^ 800CW goat anti-rabbit IgG (926–32211) were purchased from LI-COR (Lincoln, NE, United States). Protein A/G agarose gel (SC-2003) and normal mouse IgG (SC-2025) were purchased from Santa Cruz Biotechnology (Santa Cruz, CA, United States). GST-tagged mouse monoclonal antibody (AF5063) and GST-tagged purification resin (P2250) were purchased from Beyotime (Shanghai, China).

### Construction of recombinant expression plasmids

The p30 protein is encoded by the ASFV gene, CP204L. The gene was amplified by PCR using genomic DNA of the ASFV strain GZ201801 (MT496893.1) as a template. pEGFP-p30 (restriction endonuclease sites: Xho I and BamH I), pCAGGS-p30-Flag (restriction endonuclease sites: Sac I and EcoR I), and pGEX-GST-p30 (restriction endonuclease sites: BamH I and Xho I) recombinant plasmids were constructed. The host protein genes DAB2, PARP9, RPSA, OAS1, CAPG, ARPC5, and VBP1 were amplified by PCR using cDNA from porcine alveolar macrophages (PAMs) as a template. The pCAGGS-DAB2/PARP9/RPSA/OAS1/CAPG/ARPC5/VBP1-HA (restriction endonuclease sites: Sac I and EcoR I) recombinant plasmids were constructed. The plasmids were constructed *via* homologous recombination. All the recombinant plasmids were verified by sequencing. The primers used for PCR amplification are listed in Table 1.

**Table 1:** Primer sequences for PCR amplification.

Primers	Sequences (5′–3′)
pEGFP-p30-F	AGTCCGGACTCAGATCTGATTTTATTTTAAATATATCCATGAAAATGGAGGTC
pEGFP-p30-R	TTATCTAGATCCGGTTTATTTTTTTTTTAAAAGTTTAATAACCATGAGTCTTACC
pCAGGS-p30-Flag-F	CATCATTTTGGCAAAGAATTCGCCACCATGGATTTTATTTTAAATATATCCATGAAA
pCAGGS-p30-Flag-R	TGAACCGCCTCCACCGAGCTCTTTTTTTTTTAAAAGTTTAATAACCATG
pGEX-GST-p30-F	GATCTGGTTCCGCGTGGATCCGATTTTATTTTAAATATATCCATGAAAATGGAGGTCA
pGEX-GST-p30-R	GTCACGATGCGGCCGCTCGAGTTATTTTTTTTTTAAAAGTTTAATAACCATGAGTCTTACC
pCAGGS-DAB2-HA-F	CATCATTTTGGCAAAGAATTCGCCACCATGTCTAACGAAGTAGAAACGAGTGC
pCAGGS-DAB2-HA-R	TGAACCGCCTCCACCGAGCTCGGCAAAAGGATTTCCAAACGG
pCAGGS-PARP9-HA-F	CATCATTTTGGCAAAGAATTCGCCACCATGCTGACTCCCAGGCTAGAGTT
pCAGGS-PARP9-HA-R	TGAACCGCCTCCACCGAGCTCATCAACAGGGCTGCCACTTG
pCAGGS-RPSA-HA-F	CATCATTTTGGCAAAGAATTCGCCACCATGTCCGGAGCCCTCGATG
pCAGGS-RPSA-HA-R	TGAACCGCCTCCACCGAGCTCAGACCACTCAGTGGTTGTTCCTACC
pCAGGS-OAS1-HA-F	CATCATTTTGGCAAAGAATTCGCCACCATGGATACCCCTGTTAGGGACC
pCAGGS-OAS1-HA-R	TGAACCGCCTCCACCGAGCTCGATATCTTCCTCCTGTGGAGGGG
pCAGGS-CAPG-HA-F	CATCATTTTGGCAAAGAATTCGCCACCATGTACACATCCATCCCCCAGA
pCAGGS-CAPG-HA-R	TGAACCGCCTCCACCGAGCTCTTTCCAGTCCTTGAAGAATTGCT
pCAGGS-ARPC5-HA-F	CATCATTTTGGCAAAGAATTCGCCACCATGTCGAAGAACACAGTGTCGTCG
pCAGGS-ARPC5-HA-R	TGAACCGCCTCCACCGAGCTCCACGGTTTTCCTTGCAGTCAA
pCAGGS-VBP1-HA-F	CATCATTTTGGCAAAGAATTCGCCACCATGAAACAGCCTGGGAATGAGA
pCAGGS-VBP1-HA-R	TGAACCGCCTCCACCGAGCTCTGCTTTGTTCTTGGTAGAATCATCTT

### Screening of interacting proteins based on the isolated ubiquitin-mediated yeast two-hybrid system

A PAMs yeast two-hybrid cDNA library was constructed in our previous study (data not shown). In this study, the ASFV p30 protein was used as bait to screen host proteins interacting with p30 by isolating the ubiquitin-mediated yeast two-hybrid system. The bait vector plasmid pBT3-N, which carries the screening marker Leu2, was used in this study. The prey vector plasmid was pPR3-N which carried the Trp1 screening marker. First, the bait self-activation and functional verification were performed. It was determined that the bait and prey plasmids had no toxic effects on yeast. Furthermore, the function of the isolated ubiquitin system was normal. Culture conditions for library screening were also determined. The bait plasmid, pBT3-p30, was transformed into yeast NMY51 to prepare the bait strain. The PAM cDNA library was then screened by two hybridizations. An initial selection of TDO (SD/−Leu/−Trp/-His)/X-α-Gal/5 mM 3′AT medium. For further selection, positive clones on TDO/X-α-Gal/5 mM 3′AT plates were picked and transferred to QDO (SD/−Leu/−Trp/-His/−Ade)/X-α-Gal/5 mM 3′AT plates and incubated at 30°C for 4–5 days. Blue colonies on QDO/X-α-Gal/5 mM 3′AT plates were sequenced and analyzed. Sequencing results were compared using the NCBI BLAST tool to identify host proteins that may interact with the p30 protein.

### Colocalization analysis in cells

First, the cell slide was placed at the bottom of the 13 mm glass coverslips in 24-well plates. PK-15 cells were spread on a slipper and transfected when the cell density was ~75%–80%. The transfection reagent was jetOPTIMUS (PT-117-15; Polyplus, Illkirch, France). pEGFP-p30 plasmid (0.5 μg) was co-transfected with pCAGGS-DAB2/PARP9/RPSA/OAS1/CAPG/ARPC5/VBP1-HA plasmid (0.5 μg) into PK-15 cells. PAM or MA104 cells were inoculated with ASFV (MOI = 0.5). After 24 h of transfection or infection, an indirect immunofluorescence assay (IFA) was performed. The primary antibody was a mouse anti-HA monoclonal antibody, and the secondary antibody was a goat anti-mouse IgG (H + L) Alexa Fluor Plus 488 in the transfection group. The primary antibody was a mouse anti-p30 and rabbit anti-OAS1, and the secondary antibody was a goat anti-mouse IgG (H + L) Alexa Fluor Plus 488 and goat anti-rabbit IgG (H + L) Alexa Fluor Plus 594 in the infection group. In addition, nuclei were stained with DAPI. Subsequently, the cell slides were removed and fixed onto carrier slides using pine tar. Finally, the results were observed, and images were taken using a laser confocal microscope (OLYMPUS, Japan).

### Co-immunoprecipitation

HEK-293T cells were spread in 100 mm cell culture dishes. When the cell density was 75%–85%, pCAGGS-p30-Flag plasmid (5 μg) was co-transfected with pCAGGS-DAB2/PARP9/RPSA/OAS1/CAPG/ARPC5/VBP1-HA plasmid (5 μg) into the cells. After 24 h of transfection, cells were lysed for 20 min on ice with western and IP cell lysis solution (P0013, Beyotime, Shanghai, China), and whole-cell lysates were harvested. Protein A/G agarose gels (25 μl) were mixed with 400 μl of the cell lysate. Simultaneously, mouse anti-Flag monoclonal antibody and normal IgG were added for immunoprecipitation and incubated at 4°C for 2 h or overnight. The immunoprecipitated complex was washed three times with TBST buffer. Finally, the immunoprecipitated complex was resuspended in SDS-PAGE protein loading buffer and boiled at 100°C for 6–8 min. The supernatant was then used for western blot analysis.

### GST-pulldown assay

GST-p30 and GST proteins were produced by *E. coli* BL21 (DE3). The bacterial lysate supernatant was incubated with GST-tag purification resin (Beyotime) for 2 h at 4°C. The resin was washed five times with precooled PBS. Then, GST-p30 and GST gels were prepared by re-suspending the resin with PBS. The pCAGGS-DAB2/PARP9/RPSA/OAS1/CAPG/ARPC5/VBP1-HA plasmids were transfected into HEK-293T cells, and the cell lysate supernatant was collected. The GST-p30 and GST gels (40 μl) were separately incubated with the cell lysate supernatant (400 μl) for 2 h at 4°C. A control group of cell lysates transfected with the pCAGGS-HA empty vector plasmid was also established. After incubation, the gel was washed three times with cold TBST, followed by western blotting.

### Western blotting assay

SDS polyacrylamide gels were used to electrophorese protein samples, which were then transferred to PVDF membranes (IPVH00010, Millipore, Billerica, MA, United States). The membrane was blocked with 5% skimmed milk at 25°C for 2 h. Specific primary antibodies were incubated overnight at 4°C. In addition, near-infrared fluorescent dye-labeled goat anti-mouse or rabbit IgG secondary antibodies were incubated for 1 h at 25°C. The results were observed using an Odyssey two-color laser presentation system (LI-COR, Lincoln, Nebraska, United States).

### Constructing the protein–protein interaction networks

The seven validated host proteins were entered into the Protein Interrelationship Database String (version 11.5). Interaction networks were constructed using the network analyzer tool Cytoscape v.3.8.1. Briefly, the species was set to *Sus scrofa*, the maximum of 50 interaction nodes were displayed, and the rest were defaulted. Interaction relationships are represented as lines between nodes. Information on other host proteins that may interact directly or indirectly with these seven proteins was also retrieved. The results were then imported into the Cytoscape (version v3.8.1) software for annotation and visualization.

### Functional analysis of interacting proteins

A transcriptomic study on ASFV-infected porcine alveolar macrophages was performed in our laboratory. In this study, these data were used to analyze the functions of proteins that interact with p30. In addition, GO and KEGG enrichment analysis and visualization were performed using the Dr. Tom data analysis platform developed by the BGI.

## Results

### Screening of ASFV p30 interacting proteins by a yeast two-hybrid system

**Figure 1:**
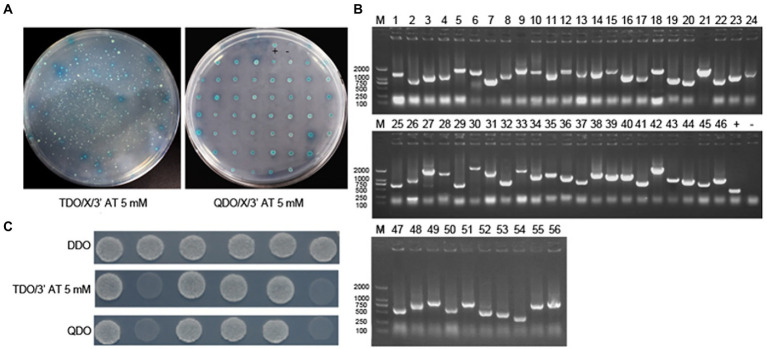
Identification of the interaction of ASFV p30 with host cellular proteins by DUALmembrane yeast two-hybrid system. **(A)** Co-transform bait and prey plasmid into NMY51, coat TDO/X/3′AT 5 mM plate, pick blue colonies, and coat QDO/X/3′AT 5 mM plate to further screen positive clones. **(B)** Agarose gel electrophoresis of PCR products of positive clone colonies. 1–53: PCR product; +: positive control (PCR using PPR3-N as template), −: negative control (PCR using water as template). **(C)** pBT3-N-CP204L (bait plasmid) was verified point-to-point with pPR3-N-Dab2 and pPR3-N-OAS1 (prey plasmid). 1: pBT3-N-CP204L and pOst1-NubI (functional verification); 2: pBT3-N-CP204L and pPR3-N (self-activation); 3: pBT3-N-CP204L and pPR3-N-Dab2 (experimental group); 4: pBT3-N-CP204L and pPR3-N-OAS1 (experimental group); +: pTSU2-APP and pNubG-Fe65 (positive control group); −: pTSU2-APP and PPR3-N (negative control group).

The bait yeast strain was transformed with a cDNA library plasmid, and positive clones were screened under pressure. The bait strain transformed by the library plasmid coated with TDO (SD/−Leu/−Trp/-His)/X/3′AT 5 mM plate showed blue colony growth, indicating that pBT3-N-Bait+pPR3-N-Prey was successfully transferred into the host strain and was non-toxic to the host strain. The blue-positive clone colonies were selected and seeded on the QDO (SD/−Leu/−Trp/-His/−Ade) /X/3′AT 5 mM plate. The growth of blue and white spots was visible, indicating that pBT3-N-Bait+pPR3-N-Prey interacted, and the reporter genes His3 and ADE2 were activated simultaneously (Figure 1A). Positive monoclonal colonies on the qdo/x/3′at 5 mm plates were selected for PCR detection and sequencing analysis. Repetitive sequences were removed after alignment. A total of 33 cell proteins may interact with the ASFV p30 protein (Figure 1B). The proteins DAB2 and OAS1 were selected for point-to-point verification. The prey plasmid (pPR3-DAB2/OAS1) and the bait plasmid (pBT3−p30) were co-transformed into yeast NMY51. The results showed that the co-transferred prey plasmid (pPR3-DAB2/OAS1) and bait plasmid (pBT3−p30) grew normally after being seeded into DDO, TDO (SD/−Leu/−Trp/-His)/5 mM 3′AT, and QDO (SD/−Leu/−Trp/-His/−Ade) nutrient-deficient plates, but no growth occurred in the self-activation and the negative control groups. This indicated that DAB2 and OAS1 interact with p30 (Figure 1C).

### Validation of interactions between PAM cellular and ASFV p30 proteins

**Figure 2:**
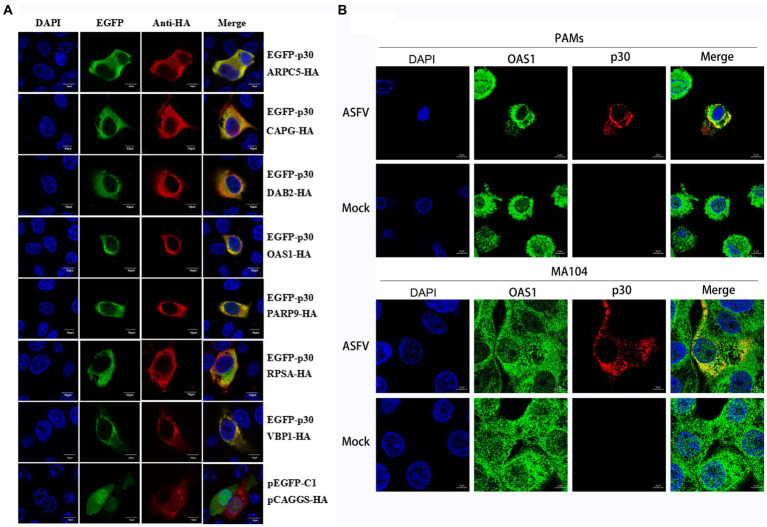
Colocalization of ASFV p30 with ARPC5, CAPG, DAB2, OAS1, PARP9, RPSA, and VBP1. **(A)** PK-15 cells were co-transfected with plasmids expressing HA-Dab2, HA-RPSA, HA-OAS1, HA-VBP1, HA-PARP9, HA-CAPG, or HA-ARPC5, and plasmids expressing EGFP-p30. Co-transfection of pEGFP-C1 and pCAGGS-HA as negative control. After 24 h, the cells were fixed with 4% PFA, stained with mouse anti-HA, and then examined by confocal microscopy. Scale bar = 10 μm. **(B)** PAM or MA104 cells were inoculated with ASFV (MOI = 0.5). After 24 h, the cells were fixed with 4% PFA, stained with mouse anti-p30 and rabbit anti-OAS1, and then examined by confocal microscopy. Scale bar = 5 μm.

**Figure 3:**
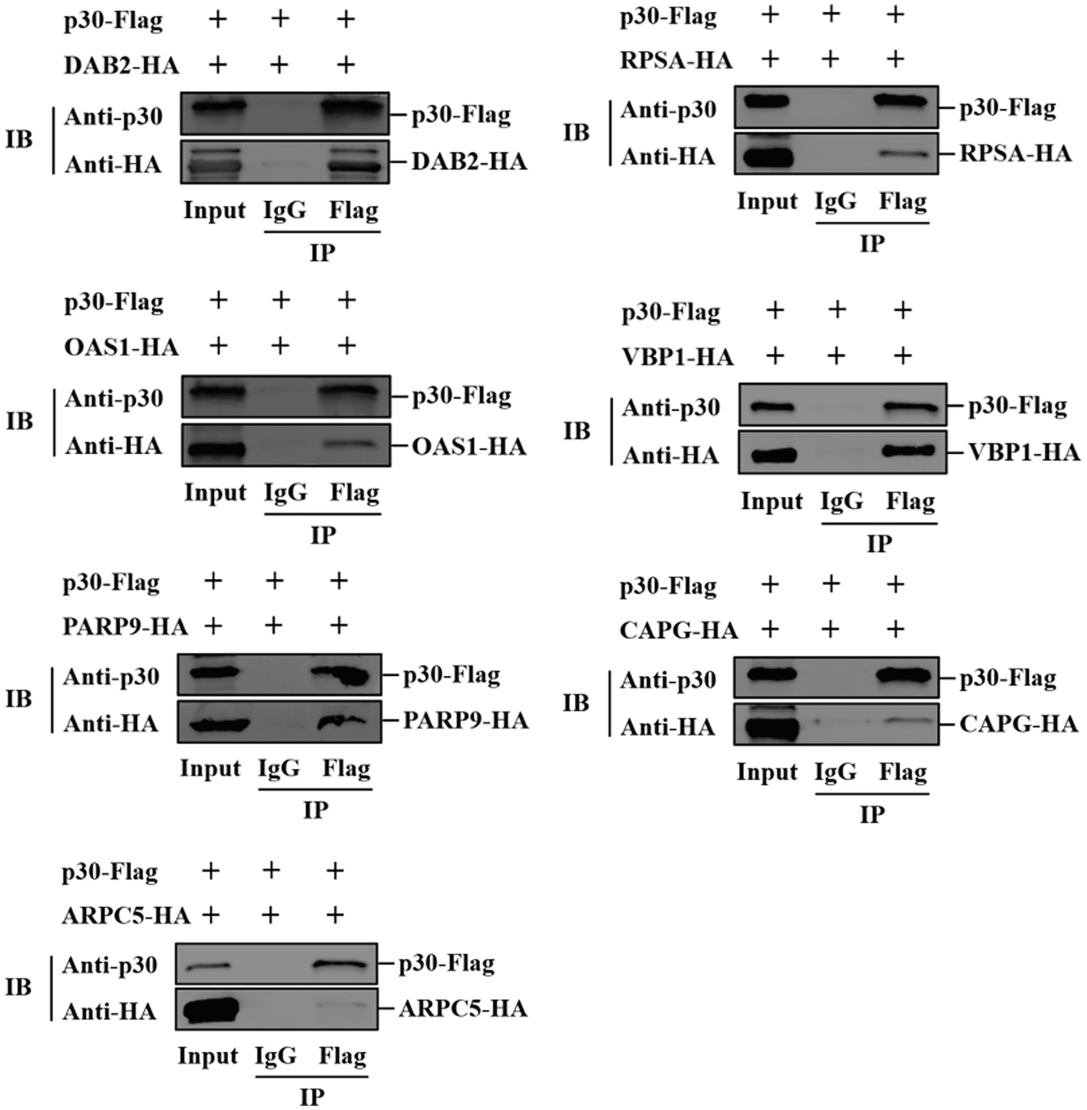
Verification of ASFV p30-cell protein interaction using Co-IP. HEK-293T cells were co-transfected with plasmids expressing HA-Dab2, HA-RPSA, HA-OAS1, HA-VBP1, HA-PARP9, HA-CAPG, or HA-ARPC5, and plasmids expressing Flag-p30. Cell lysates were immunoprecipitated with IgG and anti-Flag antibodies, followed by immunoblotting with anti-HA antibodies. The first lane indicates the input group, the second lane is the IgG control group, and the third lane is the experimental group.

**Figure 4:**
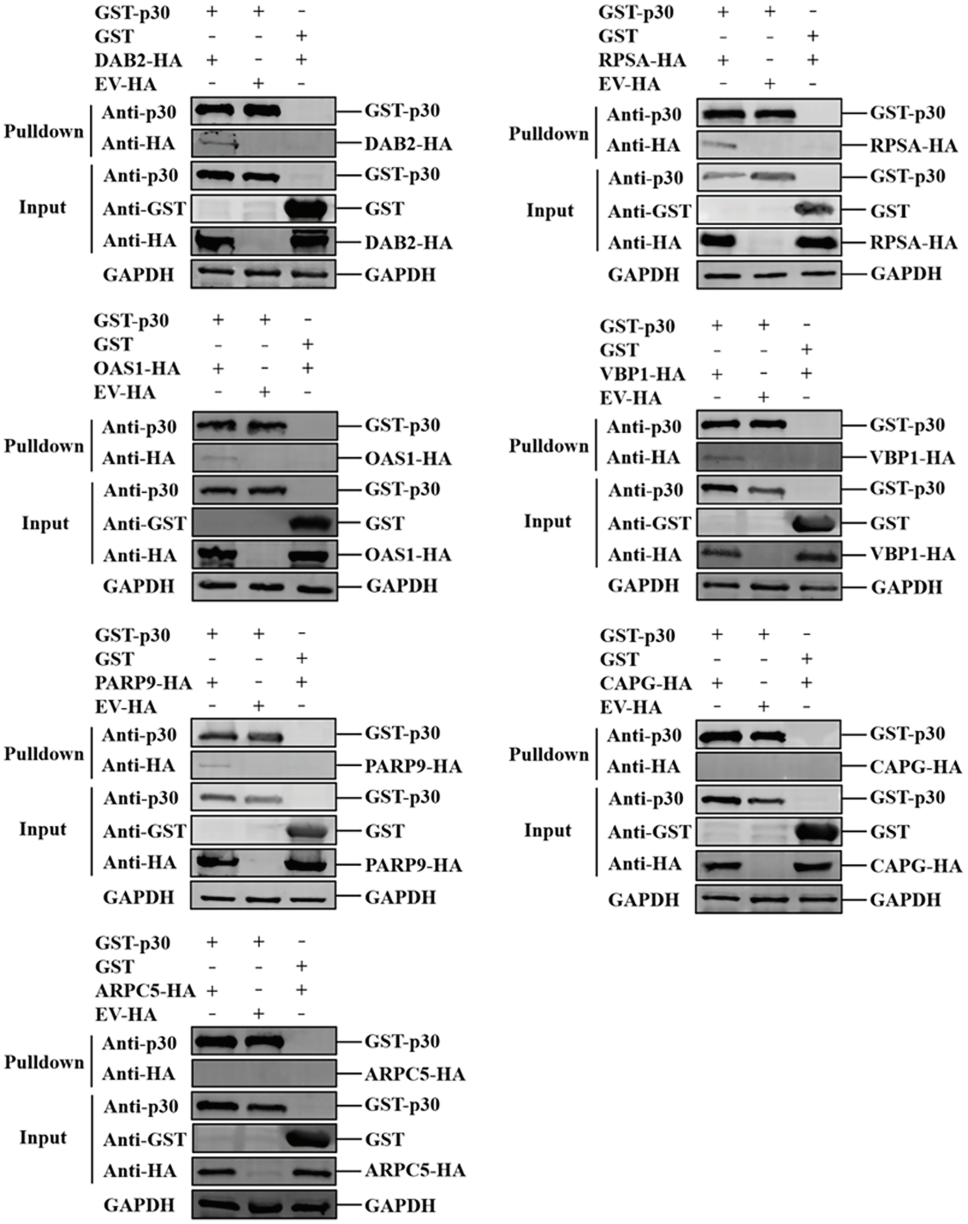
Verification of ASFV p30-cell protein interaction using GST-Pulldown assay. ASFV p30 bound to the recombinant Dab2, RPSA, OAS1, VBP1, PARP9, CAPG, or ARPC5. The GST-p30 recombinant proteins were expressed in prokaryotic cells, purified with GST beads, then incubated with lysate of HEK-293 T cells expressing HA-Dab2, HA-RPSA HA-OAS1, HA-VBP1, HA-PARP9, HA-CAPG, or HA-ARPC5. After washing with cold-PBS, the eluted complexes were determined by immunoblotting and detected with specific antibodies.

We verified the protein interaction obtained by yeast two-hybrid screening and determined whether p30 is involved in virus internalization and innate immune regulation. Seven cellular proteins were screened from p30-interacting cellular proteins related to cellular endocytosis and innate immune regulation. We constructed EGFP-tagged pEGFP-p30 plasmids with ARPC5, CAPG, DAB2, OAS1, PARP9, RPSA, and VBP1-HA plasmids and co-transfected these plasmids and p30-expressing plasmids into PK-15 cells. The results showed that the p30 protein can co-localize with ARPC5, CAPG, DAB2, OAS1, PARP9, RPSA, and VBP1 proteins (Figure 2A). In ASFV-infected PAMs and MA104 cells, we verified whether one of the interacting proteins, OAS1, co-localizes with ASFV-infected expressing p30, and the results showed that the p30 protein can co-localize with OAS1 (Figure 2B). However, the colocalization results can only prove that the proteins are localized in the same cell. To determine whether the p30 protein interacts with ARPC5, CAPG, DAB2, OAS1, PARP9, RPSA, and VBP1 proteins, we constructed a pCAGGS-p30-Flag plasmid and co-transfected HEK293T cells with the above seven cell protein plasmids for the Co-IP experiment. The results showed that the p30 protein co-immunoprecipitated with DAB2, OAS1, PARP9, RPSA, VBP1, CAPG, and ARPC5, respectively, indicating that p30 interacts with these proteins (Figure 3). We used a GST-pulldown assay to verify further the interaction between p30 and the identified proteins *in vitro*. GST-tagged p30 protein was expressed in *E. coli* and purified, HA-tagged cellular protein was expressed in HEK293T cells, and GST-p30 protein pulled down DAB2, OAS1, RPSA, PARP9, and VBP1 proteins, but not CAPG and ARPC5 proteins. This indicates that the p30 protein can directly bind and interact with DAB2, OAS1, PARP9, RPSA, and VBP1 but cannot directly bind to CAPG and ARPC5, suggesting that p30 interacts indirectly with CAPG and ARPC5 (Figure 4).

### Construction and analysis of the interaction network between the p30 and host proteins

**Figure 5:**
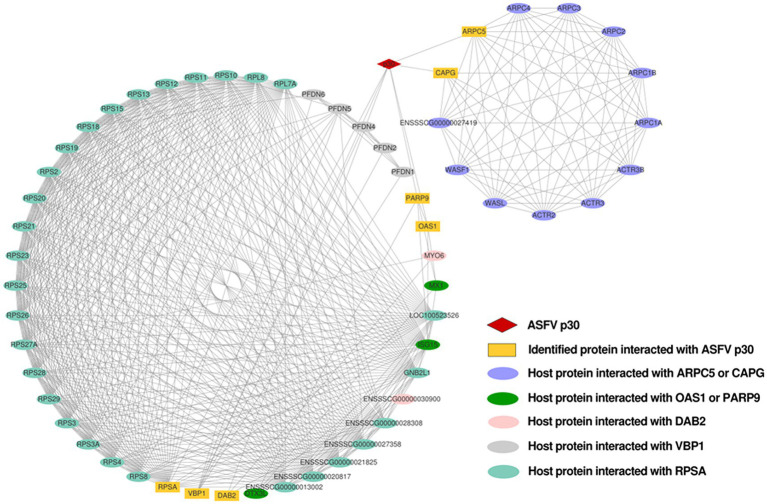
Network of interactions between ASFV p30 and host cellular proteins. Using the STRING database, a protein–protein interaction network was constructed and analyzed. Protein interactions with a total score of >0.7 from the STRING database were chosen for network development. The network analyzer tool Cytoscape v.3.8.1 was used to create and plot the map of ASFV p30-interacting proteins that interacted with the other proteins in our dataset. Straight lines between the nodes represent the interactions between proteins. Matching symbols are used to indicate distinct protein classes. NCBI gene names are used to represent proteins.

The interaction network between the ASFV p30 and host proteins was constructed using Cytoscape (Supplementary Table S1). There are 58 nodes in Figure 5. The red diamond represents the ASFV p30 protein, the yellow rectangles represent the host proteins that interact with p30, and the other ovals with different colors represent other host proteins that may interact with the above seven host proteins. The interaction between the p30 protein and 57 host proteins is connected by 573 straight lines to form an interaction network, in which RPSA, VBP1, DAB2, OAS1, and PARP9 are notable major node proteins, while ARPC5 and CAPG are minor node proteins whose host proteins interact with RPSA, VBP1, DAB2, OAS1, and PARP9 to form an interacting subnet (Figure 5).

### Enrichment analysis of p30 protein-interacting host proteins in GO function and KEGG pathway

**Figure 6:**
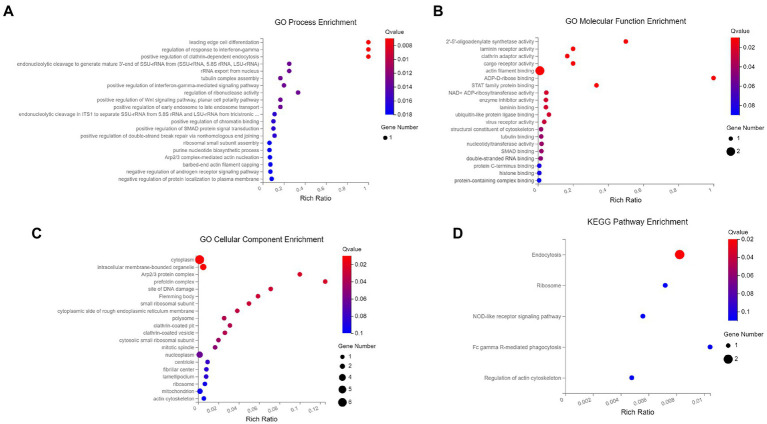
GO and KEGG pathway0 enrichment analysis. **(A)** The biological process of GO enrichment; **(B)** molecular function of GO enrichment; **(C)** cellular components of GO enrichment; **(D)** KEGG pathway enrichment. The enriched databases targeted by ASFV p30-interacting proteins. The terms that were significantly enriched (*p* < 0.05) were shown.

To analyze the functions and mechanisms of host proteins in the p30 protein interaction network, we performed GO functional enrichment and KEGG pathway enrichment analysis on seven host proteins ARPC5, CAPG, DAB2, OAS1, PARP9, RPSA, and VBP1 (Supplementary Table S2). We selected the first 20 functional enrichment results, of which the GO cellular component was mainly enriched in plasma membrane-bound organelles, Arp 2/3 complex, perforin complex, ribosomal small subunit, polynucleosome, rough endoplasmic reticulum membrane, and clathrin-coated pits and vesicles. GO molecular function is mainly enriched in 2′–5′-oligoadenylate synthase, laminin receptor, clathrin adaptor protein, cargo receptor, actin filament binding, STAT Protein family binding, NAD and ADP ribosyltransferase, and ubiquitin-like protein ligase binding. GO biological processes are mainly enriched in mediating cell differentiation and regulating interferon-gamma response, endonuclease cleavage, nuclear rRNA release, and microtubule complex assembly. Positive regulation of interferon-gamma, Wnt, planar cell polarity signaling pathways, ribonuclease activity, endosomal trafficking, SMAD protein signaling, DNA double-strand break repair, assembly of small ribosomal subunits, purine nucleotide biosynthesis, and actin nucleation was mediated by the Arp 2/3 complex. Negative regulation of androgen receptor signaling pathway and protein localization to the cell membrane. The KEGG pathway was mainly enriched in endocytosis, ribosome, NOD-like receptor signaling pathway, FcγR-mediated phagocytosis, and actin cytoskeleton regulation (Figure 6).

## Discussion

Viruses often interact with host proteins to use their cellular functions to complete virus adsorption, internalization, replication, and assembly and finally, release mature virus particles for a new round of infection. The interaction between ASFV and the host plays a crucial role in its life cycle. For example, the ASFV inner envelope proteins pE248R and pE199L can interact with the endosomal proteins such as the Niemann-Pick C type 1 (NPC1) and lysosomal membrane proteins (Lamp-1 and-2) to complete core penetration (Matamoros et al., 2020; Cuesta-Geijo et al., 2022). In addition, the interaction of ASFV p54 with cellular dynein light chain (LC8) regulates viral transport machinery (Alonso et al., 2001). ASFV I267L interacts with the E3 ubiquitin ligase Riplet, preventing the activation of RIG-I, and ultimately regulating innate immunity (Ran et al., 2022). The p30 protein is an early expressed viral protein, and studies have confirmed the importance of this protein for viral internalization (Gomez-Puertas et al., 1996). Several studies have reported using proteins involved in ASFV entry to develop an African swine fever vaccine (Gaudreault and Richt, 2019; Goatley et al., 2020). However, although many studies have reported some early events of ASFV entry and ASFV immunoregulatory functions, the role of the interaction between ASFV and host proteins in regulating these processes is still poorly understood.

**Figure 7:**
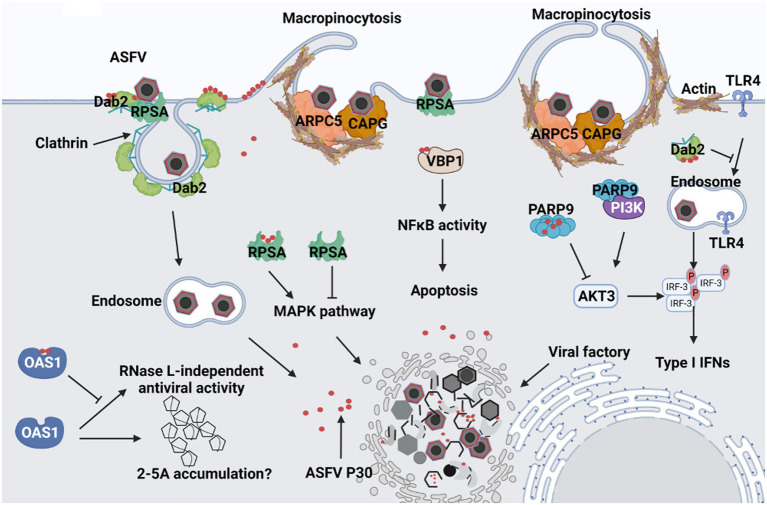
Predicted schematic model depicting the interaction between ASFV p30 and host cells. 1. After ASFV attaches to cells, it may bind to Dab2 and RPSA on the cell membrane through the structural protein p30, and then Dab2 recruits clathrin to mediate ASFV endocytosis. 2. p30 binds ARPC5 and CAPG, causing cellular actin rearrangement to trigger macropinocytosis. 3. After viral endocytosis, p30 protein may inhibit the entry of TLR4 into endosomes by recruiting Dab2 and clathrin, and the interaction between PARP9 and p30 may also inhibit the binding of PARP9 to PI3K and AKT3 activation. They ultimately inhibit IRF3 phosphorylation and reduce type I interferon production. 4. p30 may promote viral replication by interacting with RPSA to eliminate the inhibitory effect of RPSA on the MAPK pathway; p30 may prevent RNase L-dependent or independent antiviral responses by interacting with OAS1. 5. p30 can also interact with VBP1, which may enhance the activation of NF-κB and regulate the occurrence of apoptosis.

Binding to cell surface receptors is usually the first step in establishing viral infection. RPSA, also known as laminin receptor 1, is thought to be a receptor for the entry of various viruses, including flaviviruses (Thepparit and Smith, 2004; Chen et al., 2015) and alphaviruses (Wang et al., 1992; Malygin et al., 2009). Notably, in a study of the foot-and-mouth disease virus (FMDV), the interaction of the VP1 protein of FMDV with the RPSA protein abolished the inhibitory effect of RPSA on the MAPK pathway, thereby maintaining viral replication in cells (Zhu et al., 2020). Therefore, RPSA may act as an ASFV binding receptor or viral replication restriction factor, which deserves further study (Figure 7). Early studies also show that the entry of ASFV into macrophages is dependent on clathrin endocytosis (Galindo et al., 2015; Goatley et al., 2020). DAB2 assists in the recognition and recruitment of receptors to clathrin-coated pits in CME (Figliuolo et al., 2020). In addition, DAB2 binding to clathrin inhibits the entry of TLR4 into endosomes, TRIF-mediated phosphorylation of IRF3, and the expression of type I interferon (Hung et al., 2016). Therefore, we speculate that ASFV may bind to the RPSA receptor through p30 and recruit Dab2 to activate clathrin-mediated endocytosis. This binding process may also suppress the host’s innate immune response and promote viral replication.

In addition, ASFV can enter the host cells *via* macropinocytosis (Sanchez et al., 2012; Hernaez et al., 2016). Virus-induced macropinocytosis is characterized by actin-dependent plasma membrane ruffling (Mercer and Helenius, 2009). CAPG is a member of the mammalian (gelsolin) superfamily, expressed in the highest abundance in macrophages (Dabiri et al., 1992; Witke et al., 2001). Although its role in viral infection has not been reported, it is involved in macrophage membrane folding, endocytic vesicle proliferation, and signal transduction, suggesting its potential role in viral entry (Sun et al., 1995; Parikh et al., 2003). Therefore, the entry of ASFV into macrophages by inducing macropinocytosis may depend on the interaction of p30 with CAPG. We also found that actin-related protein 2/3 complex subunit 5 (ARPC5) interacts with ASFV p30 and that ARPC5 is involved in endocytosis and phagocytosis (Abella et al., 2016). Studies have shown that ARPC5 is involved in the entry of human enterovirus 71 (HEV71) into host cells *via* clathrin-mediated endocytosis (Hussain et al., 2011). The endocytic and actin cytoskeleton regulatory pathways in this study were also enriched by KEGG enrichment analysis. CAPG and ARPC5 are key molecules in the actin cytoskeleton regulatory pathway, suggesting that p30 may regulate the actin cytoskeleton rearrangement to induce macropinocytosis by binding to CAPG and ARPC5 (Figure 7).

According to the GO biological process analysis, in addition to enrichment of endosomal transport and microtubule assembly, it also includes regulation of the interferon-gamma response. The KEGG pathway enrichment analysis also revealed that in addition to the enrichment of endocytosis and actin cytoskeleton regulatory pathways, NOD-like receptor signaling pathways were also enriched. It has been suggested that p30 plays an essential role in activating innate immunity. ARP9, poly(ADP-ribosyl) polymerase 9, is a member of the poly(ADP-ribosyl) polymerase (PARPs) protein family. PARPs are involved in various biological processes such as gene transcription, cellular stress response, and antiviral innate immunity (Zhu and Zheng, 2021). In encephalomyocarditis virus (EMCV)-infected cells, PARP9 can form a complex with the E3 ubiquitin ligase DTX3L, enhancing its interaction with STAT1, promoting STAT1 nuclear translocation, activating the promoters of interferon-stimulated genes (ISGs), and ultimately inhibiting viral replication (Zhang et al., 2015). It also binds and activates the regulatory subunits of PI3K, p85, and AKT3, resulting in the phosphorylation of IRF3 and IRF7 to promote IFN-I expression (Xing et al., 2021). Therefore, we speculate that ASFV p30 regulates the host’s innate immune response by interacting with PARP9. OAS1 is a member of the 2′–5′-oligoadenylate synthase (OAS) family and plays an important role in antiviral innate immunity (Silverman, 2007; Drappier and Michiels, 2015). OAS recognizes viral dsRNA, causes ATP to generate 2′–5′adenosine (2-5A), and 2-5A activates RNase L to cleave viral RNA, preventing viral replication (Silverman, 2007). OAS/RNase L counteracts many viruses, particularly RNA viruses. However, the vaccinia virus is a large DNA virus encoding E3L, and although infection leads to accumulation of 2-5A, the RNase L cleavage process of viral dsRNA is inhibited (Rice et al., 1984; Chang et al., 1992). Similarly, the influenza virus sequesters dsRNA by encoding NS1, preventing OAS activation (Min and Krug, 2006). Based on the similarity between the vaccinia virus and ASFV, the binding of ASFV p30 to OAS1 may provide another way to escape the antiviral response of OAS/RNase L. Apoptosis is often used as a line of defense to induce innate immunity. We demonstrated that p30 interacts with VBP1. Previous studies have shown that in HBV infection, VBP1 interacts with the viral protein HBx to enhance NF-κB activation and promote apoptosis (Kim et al., 2008). Our recent study showed that deletion of MGF360 and MGF505 in ASFV can reduce the occurrence of apoptosis by inhibiting the NF-κB pathway, indicating that ASFV can regulate the NF-κB pathway by expressing related proteins that affect the apoptosis process (Gao et al., 2021). Therefore, we speculate that the interaction between p30 and VBP1 may enhance the activation of NF-κB and affect apoptosis. As a large double-stranded DNA virus, ASFV undergoes complex endocytosis and the induction of innate immunity. Therefore, future studies should further clarify the roles of cell proteins that interact with p30 in the ASFV life cycle.

## Conclusion

In this study, seven PAM cellular proteins that interact with p30 were screened and verified using a membrane yeast two-hybrid system for the first time. Through interaction network construction, GO functional analysis, and KEGG pathway analysis, these host proteins were found to be mainly involved in the endocytic pathway and the innate immune response. This study found that the interaction of p30 with RPSA, DAB2, CAPG, and ARPC5 might be involved in the viral internalization process mediated by clathrin and macropinocytosis. We also found that p30 may regulate innate immunity by interacting with innate immune regulators such as DAB2, PARP9, RPSA, OAS1, and VBP1, which has not been reported in previous ASFV studies and deserves further study. This study complements the knowledge of p30-interacting proteins and helps reveal the mechanism by which p30 is involved in viral endocytosis and host immune regulation. It also provides direction for the development of new vaccines or antiviral drugs to control ASF effectively.

## Data availability statement

The original contributions presented in the study are included in the article/Supplementary material, further inquiries can be directed to the corresponding author.

## Author contributions

LG and GZ conceived and designed the study. XioC, XiaC, SX, YL, and ZW performed the experiments. XioC, XiaC, QG, and ZH carried out the data analysis and drafted the manuscript. All authors read and approved the final manuscript.

## Funding

This research was funded by the National Key Research and Development Program of China (2021YFD1800101), the Key-Area Research and Development Program of Guangdong Province (grant number 2019B020211003), Start-up Research Project of Maoming Laboratory (2021TDQD002), and China Agriculture Research System of MOF and MARA (cars-35).

## Conflict of interest

The authors declare that the research was conducted in the absence of any commercial or financial relationships that could be construed as a potential conflict of interest.

## Publisher’s note

All claims expressed in this article are solely those of the authors and do not necessarily represent those of their affiliated organizations, or those of the publisher, the editors and the reviewers. Any product that may be evaluated in this article, or claim that may be made by its manufacturer, is not guaranteed or endorsed by the publisher.

## Supplementary material

The Supplementary Material for this article can be found online at: https://www.frontiersin.org/articles/10.3389/fmicb.2022.971888/full#supplementary-material
